# Early echocardiographic pulmonary artery measurements as prognostic indicators in left congenital diaphragmatic hernia

**DOI:** 10.1186/s12887-023-04308-3

**Published:** 2023-10-02

**Authors:** Sung Hyeon Park, Mi Jin Kim, Ha Na Lee, Jeong Min Lee, Soo Hyun Kim, Jiyoon Jeong, Byong Sop Lee, Euiseok Jung

**Affiliations:** Division of Neonatology, Department of Pediatrics, Asan Medical Center Children’s Hospital, 88, Olympic-ro 43-gil, Songpa-gu, Seoul, 05505 Korea

**Keywords:** Hernia, Diaphragmatic, Mortality, Extracorporeal membrane oxygenation, Echocardiography

## Abstract

**Background:**

To predict whether the left pulmonary artery (LPA) to the main pulmonary artery (MPA) ratio measured by echocardiography in left congenital diaphragmatic hernia (CDH) was related to death or need for extracorporeal membrane oxygenation (ECMO).

**Methods:**

This retrospective study analyzed neonates with left CDH born between 2018 and 2022 in a single tertiary medical institution. Echocardiography was performed immediately after birth. The diameter of the LPA was measured at the bifurcation, and the diameter of the MPA was measured at the maximal dimension during the systolic phase. The Nakata index, McGoon ratio, and ejection fraction (EF) were analyzed and compared with the LPA: MPA ratio as predictive values.

**Results:**

Seventy-two neonates with left CDH were included, 19 (26.4%) died or needed ECMO, and 53 (73.6%) survived without ECMO. The lower observed/expected lung-to-head ratio, lower EF, lower LPA: MPA ratio, lower RPA: MPA ratio, lower Nakata index, and lower McGoon ratio were associated with death or need for ECMO. By multivariate analysis, lower LPA: MPA ratio, RPA: MPA ratio, and Nakata index were independent postnatal risk factors for death or need for ECMO. Among the measurements, the LPA: MPA ratio had the highest area under the curve (0.957) with a sensitivity of 84.2% and specificity of 96.3% at a cut-off value of 31.2%.

**Conclusion:**

In patients with left CDH, the LPA: MPA ratio measured by echocardiography could be used as an independent postnatal predictor of death or need for ECMO.

## Introduction

Congenital diaphragmatic hernia (CDH) is one of the life-threatening congenital anomalies, occurring in 1 every 4000 live births [1]. Although advances in prenatal diagnosis and extracorporeal membrane oxygenation (ECMO) treatment have led to improved survival, the mortality rate is still approximately 30% [1, 2]. In newborn infants with CDH, the main causes of mortality are lung hypoplasia, pulmonary hypertension (PH), and cardiac dysfunction, which are intricately related [3–5]. Among these, lung hypoplasia, which carries a major mortality risk, can be predicted from prenatal measurement of the lung-to-head ratio, obtained using ultrasonography [6]. However, there has been limited research on the sonographic indices that predict clinical outcomes immediately after birth.

The diameters of branch pulmonary arteries (PAs) are correlated with lung hypoplasia in patients with CDH [7, 8]. A smaller ipsilateral PA and a larger main PA (MPA) in the prenatal period are risk factors for postnatal death and respiratory morbidity [8, 9]. A smaller branch PA and lower left PA (LPA): right PA (RPA) ratio, as measured by echocardiography on the day of birth, is associated with a need for treatment with inhaled nitric oxide (NO) [10]. The ratio between the diameters of the branch PA and the descending aorta is known as the McGoon ratio. A lower McGoon ratio is associated with a higher mortality risk in neonates with CDH [11, 12]. However, no study has yet evaluated the ratio between the diameters of the LPA and MPA for predicting CDH outcomes or the need for ECMO. Thus, this study aimed to determine whether the LPA: MPA ratio, as measured by echocardiography immediately after birth, can be used to predict mortality risk or the need for ECMO in newborn infants with left CDH.

## Materials and methods

This retrospective study analyzed a convenience sample of neonates with left CDH born between 2018 and 2022 in the neonatal intensive care unit (NICU) of Asan Medical Center, Seoul, South Korea. Right-sided CDH, preterm neonates aged < 34 gestational weeks, outborn neonates, neonates with major congenital anomalies, those with chromosomal abnormalities, and those who did not undergo echocardiography within 6 h of birth were excluded. Demographic, clinical, and echocardiographic data were collected by medical records review. Demographic data included gestational age, birthweight, sex, and small for gestational age. Clinical data included observed-to-expected lung-to-head ratio (o/e LHR) measured using the tracing method during the prenatal period, liver herniation, need for patch repair, need for ECMO, and death. For those neonates with multiple prenatal measurements of o/e LHR, the lowest value was used for analysis. The primary outcome was death or need for ECMO.

This study was approved by the Institutional Review Board (IRB) of Asan Medical Center (IRB No. 2023-0096), and the requirement for informed patient consent was waived.

### Echocardiographic assessment

**Fig. 1:**
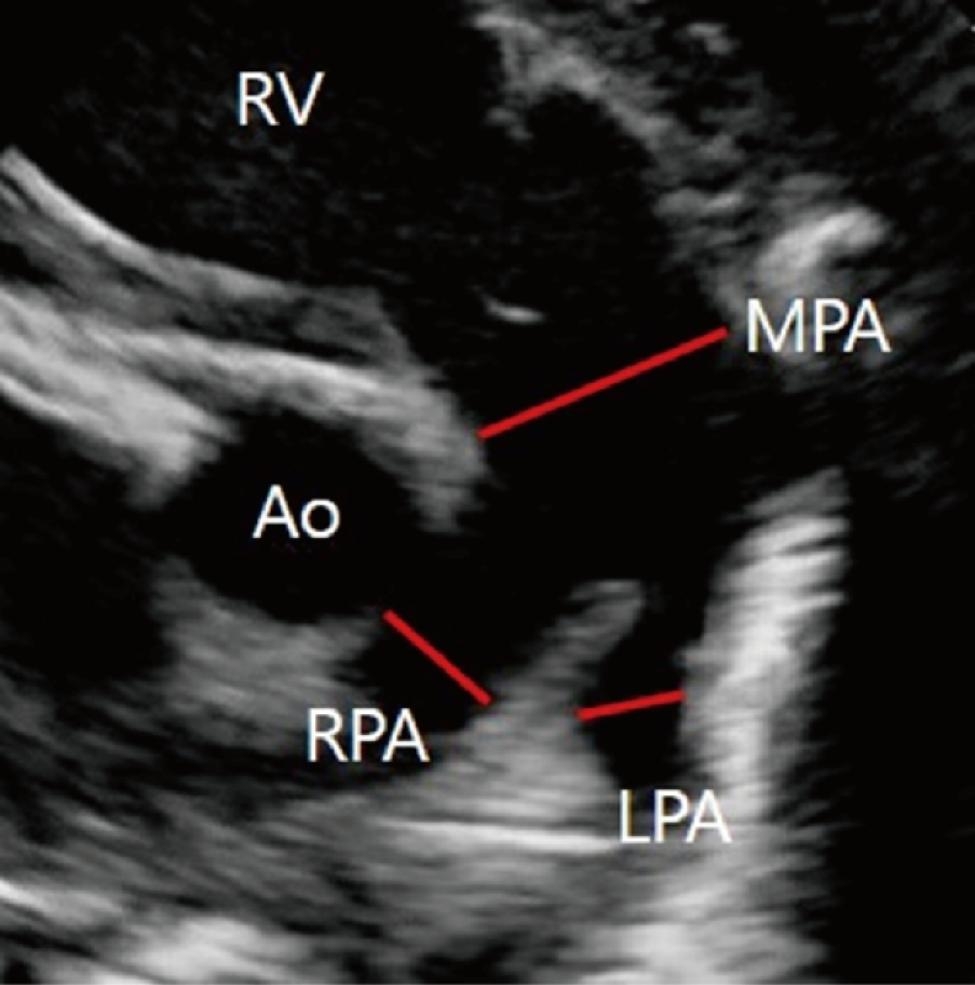
**Measuring the diameters of the main pulmonary artery (MPA) and the proximal branch pulmonary artery in a parasternal short-axis view** Abbreviations: Ao, Aorta; MPA, main pulmonary artery; RPA, right pulmonary artery; LPA, left pulmonary artery; RV, right ventricle

A single pediatric cardiologist (M.J.K) performed echocardiography on all neonates included in the study within 6 h of birth. The results were confirmed by a second pediatric cardiologist. Echocardiography was performed using a Philips EPIQ 7 system (Phillips Medical Systems, Andover, Massachusetts) with 12 MHz transducers. The echocardiographic data were collected by review of electronic medical records. In the absence of medical records, two-dimensional analysis was conducted using a TomTec® (TomTec Imaging Systems; GmbH, Unterschleissheim, Germany) on the echocardiograms. The diameters of the PAs were measured at the parasternal short-axis view (Fig. 1). The diameter of the LPA can also be measured at its origin from the MPA in a left anterior oblique or sagittal plane in a suprasternal or high left parasternal view. The diameter of the MPA was measured at its maximal dimension during systole. The proximal diameters of the RPA and LPA were measured as the origin of the vessels at the pulmonary bifurcation during systole. The diameter of the descending aorta was measured at the level of the diaphragm. The McGoon ratio was calculated as the sum of the LPA and RPA diameters divided by the diameter of the descending aorta. The Nakata index was calculated as the sum of the cross-sectional area of the LPA and RPA divided by the BSA. The cross-sectional area was calculated from the measurement of the diameter, assuming a circular vessel. The LPA: MPA ratio (%) was calculated as the diameter of the LPA divided by the diameter of the MPA. The ejection fraction (EF) was calculated in the M mode at the parasternal long-axis view. If a parasternal long-axis view was not possible, the EF was measured by biplane method at the apical four-chamber view.

### Treatment protocol

All patients were treated with a standardized CDH treatment protocol at our center. All patients were intubated immediately after birth in the delivery room, and an orogastric tube was inserted to decompress the bowel. After admission to the NICU, patients were managed by the conventional ventilator mode with permissive hypercapnia and limitation of peak inspiration pressure. High-frequency oscillatory ventilation was used in the case of refractory hypercapnia or high peak inspiratory pressures. PH was managed with inotropics including milrinone and norepinephrine. NO inhalation was employed if the left ventricular (LV) function was preserved. The criteria for administration of ECMO were as follows: (1) hypoxic respiratory failure: preductal oxygen saturation < 70% with mean airway pressure > 15 cm H_2_O or oxygenation index (OI) > 40 for > 6 h; (2) hypercapnic respiratory failure: PaCO_2_ > 70 mm Hg with pH < 7.15 with peak inspiratory pressure > 30 cm H_2_O or frequency < 7 Hz, and amplitude > 40; and (3) systemic hypotension (mean arterial pressure < 40 mm Hg) caused by PH nonresponsive to fluid and inotropic therapy. Surgical repair was performed after the stabilization of pulmonary hypertension and ventilation, or ECMO decannulation.

### Statistical analyses

The primary outcome was mortality or need for ECMO. Data were described as the mean ± standard deviation and absolute number with percentages, as appropriate. The χ2 test was used to evaluate categorical variables, and the Mann–Whitney U-test was used to compare continuous variables between groups. *P* < 0.05 was considered statistically significant. Univariate logistic regression analysis was performed, and statistically significant variables were examined by multivariate analysis. By receiver-operating characteristic curve analysis (ROC), the area under the curve (AUC) and cut-off values of variables to predict death or need for ECMO were calculated. Youden’s index was used as the cut-off value. The sensitivity, specificity, positive predictive value, and negative predictive value of the cut-off value were calculated. Based on the cut-off value, statistically significant variables were dichotomized, and the odds ratio (OR) and its corresponding 95% confidence interval (CI) for the outcome were calculated. Missing data were filled with multiple imputations by the Markov chain Monte Carlo method. Statistical analysis was conducted using IBM SPSS version 27 (IBM Corp., Armonk, NY, USA).

## Results

**Fig. 2:**
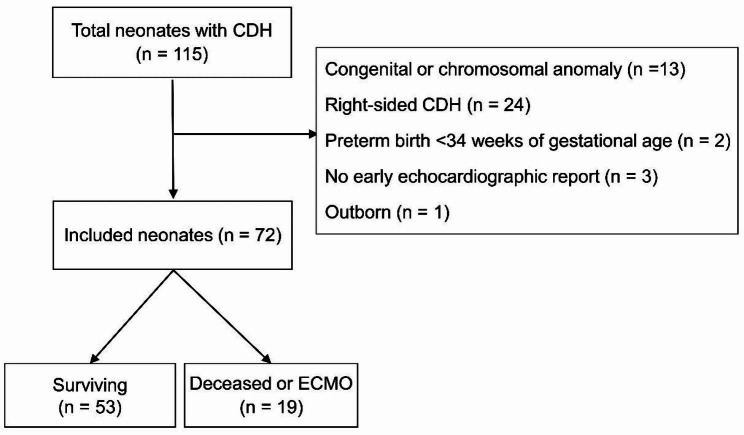
Flow diagram of the study participants

A total of 115 neonates were admitted between 2018 and 2022, and 13 patients were excluded because of major congenital or chromosomal anomalies. Twenty-four patients were excluded for having a right-sided hernia and two for preterm birth. Three patients were not enrolled because of the missing echocardiographic report immediately after birth, and one outborn patient was excluded. In total, 72 neonates were included, and 53 (73.6%) survived without ECMO (Fig. 2). The baseline characteristics of the patients are shown in Table 1. The o/e LHR and EF were lower in the deceased or ECMO group. Liver herniation and patch repair were more frequent in the deceased or ECMO group. The diameters of the RPA, and LPA were statistically smaller in the deceased or ECMO group.

**Table 1:** Baseline characteristics of the patients

	Survival (n = 53)	Deceased or ECMO (n = 19)	*P*-value
Gestational age (weeks)	38.0 ± 1.9	37.9 ± 1.3	0.405
Birth weight (g)	2947 ± 505	3011 ± 517	0.808
Male, n (%)	33 (62.3%)	10 (52.6%)	0.587
Small for gestational age, n (%)	3 (5.8%)	2 (10.5%)	0.605
o/e LHR (%)	58.7 ± 18.9	36. 4 ± 10.2	0.000
Liver herniation, n (%)	10 (18.9%)	15 (78.9%)	0.000
Patch repair, n (%)	20 (37.7%)	12/12 (100%)	0.000
EF (%)	70.4 ± 11.4	39.2 ± 16.47	0.000
MPA diameter (mm)	8.00 ± 1.14	8.86 ± 1.17	0.002
RPA diameter (mm)	3.98 ± 0.75	3.27 ± 0.64	0.000
LPA diameter (mm)	3.53 ± 0.65	2.34 ± 0.52	0.000

Data were presented as mean ± standard deviation or number (percentage)

ECMO, extracorporeal membrane oxygenation; EF, ejection fraction; LPA, left pulmonary artery; MPA, main pulmonary artery; o/e LHR, observed/expected lung-to-head ratio; RPA, right pulmonary artery

The risk factors for death or need for ECMO in neonates with left CDH are shown in Table 2. The o/e LHR and liver herniation were associated with death or need for ECMO. In the univariate analysis, the EF, LPA: MPA ratio, RPA: MPA ratio, Nakata index, and McGoon ratio were statistically significant variables for predicting outcomes. The LPA: MPA ratio, RPA: MPA ratio, and Nakata index remained significant predictors even after adjustment for gestational age (GA), liver herniation, and o/e LHR (Table 3).

**Table 2:** Risk factors for death or need for ECMO in neonates with left congenital diaphragmatic hernia

	*P*-value	OR
Gestational age (weeks)	0.718	0.949 (0.713–1.262)
Birthweight (g)	0.635	1.000 (0.999–1.001)
Male, n (%)	0.464	0.673 (0.234–1.940)
Liver herniation	0.000	16.125 (4.395–59.166)
o/e LHR (%)	0.000	0.846 (0.776–0.922)
EF (%)	0.000	0.859 (0.795–0.929)
LPA: MPA ratio (%)	0.000	0.814 (0.733–0.904)
RPA: MPA ratio (%)	0.001	0.859 (0.790–0.933)
LPA: RPA ratio (%)	0.003	0.942 (0.906–0.980)
Nakata index	0.000	0.948 (0.922–0.975)
McGoon ratio	0.037	0.103 (0.012–0.870)

Abbreviations: ECMO, extracorporeal membrane oxygenation; EF, ejection fraction; LPA, left pulmonary artery; MPA, main pulmonary artery; o/e LHR, observed/expected lung-to-head ratio; OR, odds ratio; RPA, right pulmonary artery

**Table 3:** Risk factors for death or need for ECMO by multivariate analysis

	*P*-value	OR
EF (%)	0.071	0.766 (0.573–1.023)
LPA: MPA ratio (%)	0.008	0.716 (0.559–0.916)
RPA: MPA ratio (%)	0.022	0.884 (0.795–0.983)
LPA: RPA ratio (%)	0.111	0.953 (0.898–1.011)
Nakata index	0.012	0.936 (0.889–0.986)
McGoon ratio	0.279	0.164 (0.006–4.322)

*Adjusted by gestational age, liver herniation, o/e LHR

Abbreviations: ECMO, extracorporeal membrane oxygenation; EF, ejection fraction; LPA, left pulmonary artery; MPA, main pulmonary artery; o/e LHR, observed/expected lung-to-head ratio; OR, odds ratio; RPA, right pulmonary artery

**Fig. 3:**
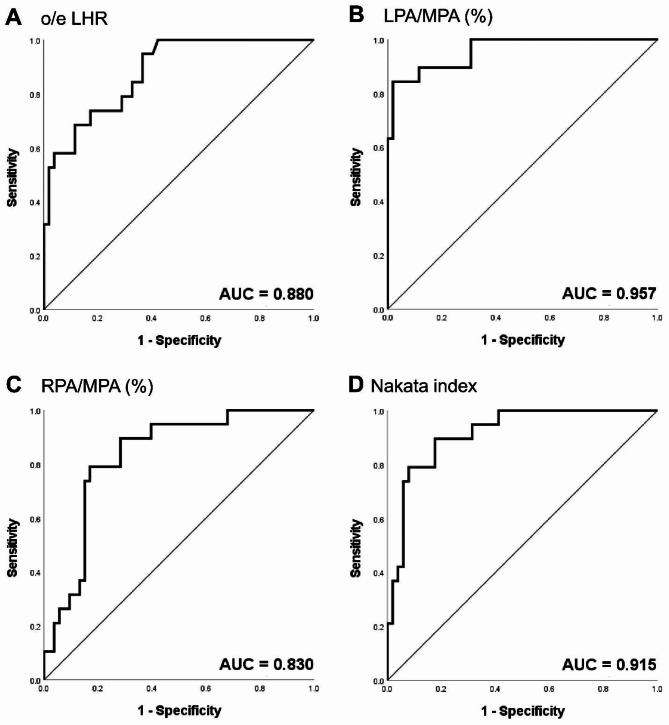
**Receiver-operating characteristic curves of variables for predicting outcomes** Abbreviations: AUC, area under the curve; LPA, left pulmonary artery; MPA, main pulmonary artery; o/e LHR, observed/expected lung-to-head ratio; RPA, right pulmonary artery

The results of the ROC analysis and the AUC are illustrated in Fig. 3. Four variables were selected independent predictors for this analysis: o/e LHR, which is a widely used prenatal predictor; LPA: MPA ratio; RPA: MPA ratio; and Nakata index. The AUC of the o/e LHR was 0.880 (*P* = 0.000; 95% CI 0.798–0.962). The highest AUC was observed for the LPA: MPA ratio at 0.957 (*P* = 0.000; 95% CI 0.909–1.000), followed by the RPA: MPA at 0.830 (*P* = 0.000; 95% CI 0.731–0.930), and the Nakata index, at 0.915 (*P* = 0.000; 95% CI 0.848–0.983). The cut-off values of the variables were 49.9%, 31.2%, 40.1%, and 90, respectively (Table 4). The variables were dichotomized by the cut-off values, and the ORs of the o/e LHR, LPA: MPA ratio, RPA: MPA ratio, and Nakata index were 14.76, 272, 18.33, and 39.67, respectively (Table 5). Following adjustment for several factors as previously done, the LPA/MPA ratio showed a highest OR.

**Table 4:** Cut-off value of death or need for ECMO using variables

	Cut-off value	Sensitivity	Specificity	Positive predictive value	Negative predictive value
o/e LHR (%)	49.9	94.7	63.5	48.7	97.1
LPA: MPA ratio (%)	31.2	84.2	96.3	88.9	94.4
RPA: MPA ratio (%)	40.1	79.0	83.0	62.5	91.7
Nakata index	90	89.5	80.8	63.0	95.5

Abbreviations: ECMO, extracorporeal membrane oxygenation; LPA, left pulmonary artery; MPA, main pulmonary artery; o/e LHR, observed/expected lung-to-head ratio; RPA, right pulmonary artery

**Table 5:** Odds ratio of dichotomized variables based on the cut-off value for death or need for ECMO

	Unadjusted		Adjusted*	
	*P*-value	OR	*P*-value	OR
o/e LHR (%)	0.001	14.76 (3.07–70.97)		
LPA: MPA ratio (%)	0.000	272 (26.42–2800.68)	0.000	187.24 (10.13–3459.9)
RPA: MPA ratio (%)	0.000	18.33 (4.92–68.32)	0.002	18.3 (2.88–116.61)
Nakata index	0.000	39.67 (7.75–202.96)	0.001	24.75 (3.63–168.6)

*Adjusted by gestational age, liver herniation, and o/e LHR

Abbreviations: ECMO, extracorporeal membrane oxygenation; EF, ejection fraction; LPA, left pulmonary artery; MPA, main pulmonary artery; o/e LHR, observed/expected lung-to-head ratio; OR, odds ratio; RPA, right pulmonary artery

## Discussion

Analyses of data from the CDH cohort of a single institution revealed the following major findings. First, early echocardiographic measurement of the LPA: MPA ratio was found to be an independent predictor of mortality and the need for ECMO in newborn infants with left CDH. Second, the o/e LHR value was valid as a risk factor, which was in line with previous studies [6].

In the present study, both the LPA: MPA ratio and the o/e LHR were found to be useful predictors of mortality risk and the need for ECMO. However, the AUC showed a slightly superior value for the LPA: MPA ratio. The o/e LHR is the most widely used prenatal predictor of postnatal outcomes but it has some limitations compared to postnatal assessment. It is measured during the prenatal period, which means there is a time interval between measurement and postnatal management. In addition, although the o/e LHR does not change with advancing gestational age [6, 13], in cases of multiple evaluation, the minimal value is known to be the most accurate and significant predictor of outcomes [14], so single measurement of the o/e LHR might be less accurate. Furthermore, 30–40% of patients with CDH were not diagnosed prenatally; therefore, their o/e LHR was not available [15, 16]. In this case, evaluating the severity and preparing for treatment immediately after birth are difficult; however, this can be solved by measuring the LPA: MPA ratio postnatally. In addition, it is relatively easy for clinicians to measure the diameters of the LPA and MPA using point-of-care echocardiography.

In this study, the deceased or ECMO group had smaller LPA and larger MPA than the surviving group, which is consistent with the findings of a previous study [10]. The dilation of MPA is due to decreased LPA flow in the left CDH [8] and persistent PH [17]. We chose the LPA: MPA ratio as an outcome predictor, not the diameter of the LPA in the left CDH, because of the following reason. The diameters of the PA might correlate with the body size of the patient [18], any effect of this correlation can be minimized because the LPA: MPA ratio is the proportion of PA diameters.

The Nakata index was also an independent predictor of mortality or need for ECMO. The Nakata index, also known as the PA index, is widely used to predict surgical outcomes in congenital heart disease [19]. In a previous study, the Nakata index was a useful predictor of disease severity and mortality in patients with CDH, and its cut-off value was 90 [20]. The AUC of the Nakata index was superior to that of the o/e LHR (0.915 vs. 0.880). The cut-off value was 90, similar to that reported in a previous study. The LPA: MPA ratio was superior to the Nakata index for predicting outcomes in patients with left CDH by the multivariate analysis and AUC. This is due to the difference between the two parameters. The Nakata index includes the cross-sectional area of the RPA but not the MPA. A small ipsilateral PA when compared with the contralateral PA is associated with poor outcomes [8, 10]. In the present study, a lower LPA: RPA ratio was statistically significant in the univariate analysis. Therefore, the use of the LPA diameter alone without the RPA diameter can predict poor outcomes more accurately. In addition, dilated MPA is associated with poor outcomes [8]; thus, the LPA: MPA ratio can be superior to the Nakata index for predicting mortality in patients with left CDH.

The McGoon ratio was a statistically significant factor in predicting outcomes in the univariate analysis, but not in the multivariate analysis. Previous studies using the McGoon ratio as a predictor of outcome have shown controversial results. Three studies have shown that a lower McGoon ratio is associated with poor outcomes [11, 12, 20]; however, in another study, no association was found between the McGoon ratio and the outcome of patients with CDH [21]. Cardiac dysfunction is associated with poor outcomes [4, 21, 22]. Owing to the decreased cardiac output, the aorta size of patients with poor outcomes might be smaller than that of patients with good outcomes. In this study, the abdominal aorta of the ECMO or deceased group was smaller than those of the surviving groups, although not statistically significant. The McGoon ratio is the sum of RPA and LPA diameters divided by the abdominal aorta, so a relatively small aorta could make the McGoon ratio a less significant predictor of mortality.

In this study, LV dysfunction was predictive of mortality and need for ECMO, although not significant in the multivariate analysis. In a previous study, LV dysfunction was an independent and stronger predictor of poor outcomes than right ventricular (RV) dysfunction [4, 22]. In patients with CDH, compression of the left ventricle by the enlarged right ventricle and herniated abdominal contents can decrease LV filling, and RV dysfunction might cause LV dysfunction due to ventricular interdependence [23, 24]. LV dysfunction can cause systemic hypotension, hypoxia, and acidosis, and this might lead to poor outcomes including mortality or need for ECMO.

This study used EF as a indicator of LV dysfunction, but EF has several limitations for this use in patients with CDH. First, EF is measured with a two-dimentional method that assumes the LV is cylinder shaped [25], but in CDH, due to compression of the LV by the RV and herniated organs, the LV might not be cylinder shaped. Second, EF is the ratio of the end-systolic volume (ESV) to the end diastolic volume (EDV), which implies that a reduced ESV and EDV owing to LV compression might not necessarily lead to a decrease in EF. Third, conditions like patent ductus arteriosus could potentially increase EF [26]. For these reasons, EF alone may not be a good marker of LV function. More prospective studies are needed to validate LV dysfunction and the outcomes of CDH.

This study had several limitations. First, this is a retrospective, single-center study that analyzed a relatively small sample size. However, data were collected through a short-period cohort with a consistent strategy; thus, the outcome was highly reliable. Second, a review of echocardiography results was not blinded; however, due to the study’s retrospective nature, the measurement of PA diameters could be unbiased. Third, although the technique of measuring diameters could be different between observers, echocardiography was performed by one pediatric cardiologist and double-checked by another pediatric cardiologist. Fourth, some data were missing, and this was minimized by filling with multiple imputations. All PAs diameters were available, and only a few data of the EF and McGoon ratio were missed. Therefore, this might not affect our main result of the LPA: MPA ratio as a predictor of left CDH outcomes.

In conclusion, the LPA: MPA ratio was found to be the best early echocardiography index among the PA diameter variables for predicting mortality and the need for ECMO in newborn infants with CDH. Our study suggests that measuring PA diameters immediately after birth is a simple and useful means of management preparation in CDH, particularly for patients who are not prenatally diagnosed.

## Data Availability

The datasets used and/or analysed during the current study are available from the corresponding author on reasonable request.

## Abbreviations

AUC: area under the curve
CDH: congenital diaphragmatic hernia
ECMO: extracorporeal membrane oxygenation
EF: ejection fraction
LPA: left pulmonary artery
MPA: main pulmonary artery
o/e LHR: observed/expected lung-to-head ratio
OR: odds ratio
RPA: right pulmonary artery
ROC: receiver-operating characteristic
