# Correction: Early echocardiographic pulmonary artery measurements as prognostic indicators in left congenital diaphragmatic hernia

**DOI:** 10.1186/s12887-024-04863-3

**Published:** 2024-06-01

**Authors:** Sung Hyeon Park, Mi Jin Kim, Ha Na Lee, Jeong Min Lee, Soo Hyun Kim, Jiyoon Jeong, Byong Sop Lee, Euiseok Jung

**Affiliations:** Division of Neonatology, Department of Pediatrics, Asan Medical Center Children’s Hospital, University of Ulsan College of Medicine, 88, Olympic-ro 43-gil, Songpa-gu, Seoul, 05505 Korea

**Correction:** ***BMC Pediatr*** **23, 499 (2023)**

10.1186/s12887-023-04308-3

Following the publication of the original article [1], the authors would like to update the affiliation of the author Euiseok Jung.

Euiseok Jung’s affiliation currently reads:

Division of Neonatology, Department of Pediatrics, Asan Medical Center Children’s Hospital, 88, Olympic-ro 43-gil, Songpa-gu, Seoul 05505, Korea

Euiseok Jung’s affiliation should read:

Division of Neonatology, Department of Pediatrics, Asan Medical Center Children’s Hospital, University of Ulsan College of Medicine, 88, Olympic-ro 43-gil, Songpa-gu, Seoul, 05505, Korea
